# αB-Crystallin Protects Against Cisplatin-Induced Nephrotoxicity by Modulating Apoptosis In Vivo and In Vitro

**DOI:** 10.3390/cimb48070667

**Published:** 2026-06-29

**Authors:** Sylia Ardache, Shu Tang, Endong Bao

**Affiliations:** College of Veterinary Medicine, Nanjing Agricultural University, Nanjing 210095, China; syliard@stu.njau.edu.cn

**Keywords:** cisplatin nephrotoxicity, αB-crystallin, apoptosis, pathways

## Abstract

Cisplatin (CP) chemotherapy is limited by nephrotoxicity, primarily involving tubular epithelial cell apoptosis. αB-crystallin (CryAB) is a small heat shock protein that plays a cytoprotective role in stressed kidneys but can also promote tumor progression. Its precise role and molecular mechanisms in CP-induced kidney injury remain largely unclear. This study highlighted the function of CryAB and its regulatory pathways in CP nephrotoxicity by employing in vitro models of rat renal tubular epithelial cells (NRK-52E) with CryAB gene knockdown/overexpression, and in vivo models of CryAB knockout/wild-type mice, followed by CP treatment. Apoptosis and key signaling pathways (NF-κB, MAPK, AKT) were evaluated in this study. The results indicated that CP treatment (20 µM) significantly upregulated CryAB expression in renal cells (*p* < 0.01) and triggered both apoptosis and MAPK activation. CryAB deficiency sensitized cells and mice to CP, exacerbating renal dysfunction, tubular injury, and apoptosis, as evidenced by increased Bax, cyt *c* release, and caspase-3 cleavage. Conversely, CryAB overexpression attenuated these effects. Furthermore, our findings suggest that the lack of CryAB favors the cytoplasmic retention of NF-κB, and that CryAB status can influence MAPK signaling, pointing to a potential regulatory loop. Additionally, CP-induced AKT phosphorylation was diminished in CryAB-deficient models. Therefore, CryAB may exert a cytoprotective role in CP nephrotoxicity, potentially mitigating tubular apoptosis by modulating the mitochondrial apoptotic pathway, supporting NF-κB-mediated survival signaling, and cross-talking with MAPK and AKT pathways. Our findings suggest that CryAB serves as an important regulator of renal cell fate and a potential therapeutic target for mitigating CP-induced kidney injury.

## 1. Introduction

Cisplatin (CP) is widely used as a chemotherapeutic agent against various cancers, including testicular, ovarian, and bladder carcinomas [1,2,3]. Nonetheless, its clinical application is significantly limited by dose-dependent nephrotoxicity, which frequently results in acute kidney injury (AKI) in both patients and experimental animals [4]. Given its status as a foundational first-line chemotherapeutic drug, minimizing the adverse effects of CP through novel combination regimens has become a primary focus of recent strategies [2,5]. Renal damage is primarily attributed to CP accumulation in tubular cells, which induces cellular stress and triggers cell death through multiple pathways. Following transport via copper transporter CTR1 and organic cation transporter OCT2, CP is intracellularly activated to form DNA platinum adducts. These adducts initiate a cascade of events, including inflammation, mitochondrial dysfunction, and oxidative stress [6,7], with apoptosis being recognized as a major mechanism of tubular cell death [8]. One of the mechanisms of cellular defense in response to stress “heat shock response” [9]. The small heat shock protein αB-crystallin (CryAB), among the proteins in the renal medulla, accounts for around 2% of all proteins and thus plays an important role in maintaining cell integrity during osmotic stressors [10]. Its expression is upregulated by nephrotoxic agents, demonstrating cytoprotective properties against tubular apoptosis, a mechanism mediated in part through heat shock factor 1 (HSF1) [11]. Additionally, CryAB is implicated in modulating inflammatory responses and repair processes, underscoring its importance in the kidney’s adaptation to injury [12]. Recent studies emphasize its role in coping with cellular stress through chaperoning misfolded proteins and inhibiting apoptosis [13]. By stabilizing mitochondrial function and mitigating ROS production, CryAB counteracts CP-induced oxidative stress [11], and its chaperone activity promotes the refolding of denatured proteins, supporting cell survival. Upregulation of CryAB has been linked to enhanced cell viability and suppressed apoptotic signaling [14]. However, its role is complex: while protecting cells, its overexpression has been associated with tumor progression in renal cell carcinoma, revealing a paradoxical function in both tissue repair and malignancy [15,16]. This duality highlights the need to clarify the precise function of CryAB and its modulation of cellular stress response during CP-induced renal damage.

Three main mammalian MAPK subfamilies are recognized, ERK, JNK, and p38 MAPK, which are stress-responsive kinases [17]. CP treatment has been shown to activate this signaling cascade in renal tubular injury. While ERK1/2 activation is time- and dose-dependent, though its level varies across cell types and stress conditions, with some cells exhibiting minimal activity [17,18], a phenomenon observed, for instance, in CP-resistant HeLa cell variants [19]. p38 MAPK is also activated by CP, with sensitive cells displaying sustained activation (8–12 h) and resistant cells demonstrating a transient response (1–3 h); impaired p38 function has been linked to a resistant phenotype [20,21]. CP exposure typically triggers a delayed and persistent JNK response. Notably, the expression of MAP kinase phosphatase 1 (MKP 1) debilitates JNK and p38 activation, correlating with increased cell survival, although MKP 1 itself is only modestly activated by CP [22,23].

In light of this background, the current investigation was intended to examine the role of CryAB in CP-induced renal toxicity, focusing primarily on its anti-apoptotic action and the involvement of the MAPK/AKT pathways in mitigating mitochondrial apoptosis and nephrotoxicity. A CP-induced renal injury model was established, using loss- and gain-of-function strategies for CryAB in both mouse kidney and cultured NRK-52E rat kidney cells.

## 2. Materials and Methods

### 2.1. Reagents

The NRK-52E normal rat kidney epithelial cell line used in this study was supplied by the ATCC cell bank (Manassas, VA, USA). The cell culture media (DMEM and FBS) and supplements (Opti-MEM) were obtained from Gibco (Waltham, MA, USA). The transfection agent Lipofectamine 3000 was purchased from Invitrogen (Waltham, MA, USA), Zeocin selection reagent was purchased from Invitrogen (Carlsbad, CA, USA), and puromycin was obtained from Macklin Biochemical Technology (Shanghai, China). CP was purchased from Sigma-Aldrich (St. Louis, MO, USA), and Cell Counting Kit-8 assay kits were provided by Beyotime Biotechnology (Shanghai, China). Immunohistochemistry (SP rabbit and mouse HRP) kits were obtained from CWBIO (Taizhou, China), and apoptosis kits TUNEL were obtained from KeyGEN BioTECH (Nanjing, China). The lentiviral vector expressing short hairpin RNA (shRNA) targeting CryAB was constructed by HANBIO Biotechnology (Shanghai, China); Clone ID: HBLV-r-CryAB-shRNA1-ZsGreen-PURO; Cat No: JY20230302RFF-LV01. And No: HH20220920RFF-SI01.

Antibodies used in this study included: CryAB was obtained from ENZO (Farmingdale, NY, USA); BAX was purchased from Boster Bio (Pleasanton, CA, USA); Caspase-3 (Casp-3), BCL-2, NF-κB, p-P65, Hsp 70, p-P38, and goat HRP conjugated anti-mouse and anti-rabbit secondary antibodies were obtained from ABclonal (Woburn, MA, USA). Cleaved Casp-3, cyt *c*, ERK, p-ERK, AKT, P38, p-AKT, and JNK were purchased from Cell Signaling Technology (Danvers, MA, USA). GAPDH was obtained from Abcam (Waltham, MA, USA).

### 2.2. Experimental Animals

Eight-week-old wild-type C57BL/6J mice (WT) were sourced from the Experimental Animal Center of Yangzhou University. Systemic CryAB knockout mice (KO) were provided by the Nanjing Institute of Biomedicine at Nanjing University. All experimental procedures involving animals followed protocols authorized by the Institutional Animal Care and Use Committee of Nanjing Agricultural University and adhered to applicable ethical standards for animal research (PZ2020094, 31 August 2020).

### 2.3. Cell Culture and Transfection

NRK-52E rat kidney epithelial cells were cultured in Dulbecco’s Modified Eagle Medium supplemented with 5% fetal bovine serum at 37 °C in a humidified atmosphere with 5% CO_2_. For experimental use, cells were seeded into 35 mm dishes at an initial density of 1 × 10^6^ cells per dish and incubated until they reached 80–90% confluency.

To knock down CryAB expression, cells were cultured in six-well plates and co-transfected for 24 h with either an shRNA vector targeting CryAB (HBLV-r-CryAB-shRNA1-ZsGreen-PURO) or a non-targeting control shRNA vector (pGFP-V-RS). Both constructs contained a puromycin resistance marker. Transfection was carried out with Lipofectamine 3000, adhering to the manufacturer’s guidelines. After transfection, the positive cells were screened by adding 8 μg/mL puromycin for 3 days, after which the puromycin concentration was lowered and maintained for an additional 5 days. The resulting CryAB knockdown cells and scramble shRNA control cells were expanded, subcultured, and stored at −80 °C for subsequent Western blot analysis and downstream experiments. The shRNA oligonucleotide sequences are detailed in Table 1.

### 2.4. Animal Experimental Designs

WT and KO mice were maintained in specific pathogen-free (SPF) facilities with a controlled 12 h light/dark cycle, stable temperature, and regulated humidity. Prior to treatment, animals were randomly divided into their respective experimental groups without selection or sorting criteria. To account for variation in body weight, a balanced sex distribution was maintained by keeping 3 male and 3 female mice per designated group. The entire experimental study was replicated into three independent biological timeline trials.

WT mice were allocated into 4 experimental groups (6 mice per group): a control group (WT-Control), and three treatment groups receiving CP at doses of 10 mg/kg body weight (WT-CP10), 20 mg/kg (WT-CP20), and 30 mg/kg (WT-CP30). Similarly, KO mice were assigned to equivalent experimental groups (*n* = 6 mice per group): KO-Control, KO-CP10, KO-CP20, and KO-CP30.

To establish kidney damage, mice were given one intraperitoneal (i.p.) dose of CP corresponding to their assigned group. Control mice received an equivalent volume of 0.9% saline instead. To assess model kinetics, physical symptoms and survival were monitored daily across experimental groups; changes were evaluated through body weight measurement (individual animal body weights were recorded longitudinally at four intervals: prior to injection or 0 h, 24 h, 48 h, 72 h post injection), survival tracking, and qualitative visual observation of general body motor and body posture twice daily. Animal exclusion criterion was restricted to mortality before the scheduled day for experimental euthanasia.

All mice were humanely euthanized 72 h (3 days) after CP treatment by cervical dislocation to collect urine and blood samples, after which the kidneys were harvested for further study. Animal mortality was evaluated as a pre-specified experimental endpoint rather than being treated as data exclusion. For downstream histological and biological assays, data were collected from all successfully surviving animals.

To minimize experimental bias, sample collection and subsequent experimental analyses were conducted in a blinded manner till completion of final data generation.

### 2.5. Renal Function Analysis

Blood samples were collected from the retro-orbital plexus of mice and centrifuged at 1000× *g* for 10 min at 4 °C to obtain serum. Serum levels of creatinine (Cr), blood urea nitrogen (BUN), alkaline phosphatase (ALP), and lactate dehydrogenase (LDH) were measured using a chemistry analyzer (Mindray BS 240VET, Shenzhen, China). Urinary kidney injury molecule 1 (uKIM-1) levels were quantified using a commercial mouse KIM-1 enzyme-linked immunosorbent assay kit (CWBIO, Taizhou, China) following the respective manufacturer’s protocol.

### 2.6. Cell Experimental Designs and the Cell Viability Assay

After seeding the cells at a density of 5 × 10^3^ cells per well, the cells were cultured for 24 h under the conditions described above and then treated with CP at (0, 10, 20, 50, or 100 µM) for an additional 24 h. To determine cell viability, 10 µL of CCK-8 solution was added to each well, and absorbance was evaluated against untreated cells (containing cells, media, and CCK-8) and cell-free blank wells containing equivalent volumes of media and CCK-8 solution (negative controls). After incubating at 37 °C in 5% CO_2_ for 1 h, the optical density (OD) of each well was measured at 450 nm using a scanning multiwell spectrophotometer (Tecan, Switzerland). The following formula was used to estimate the relative cell viability: Relative cell viability (%) = [(OD_450_ treated − OD_450_ blank)/(OD_450_ control − OD_450_ blank)] × 100.

### 2.7. Histopathological and Cytopathological Observation After CP Treatment

The kidney tissue samples were formalin-fixed and embedded in paraffin. Paraffin sections (4 μm) were subjected to standard histological processing, including deparaffinization and rehydration, and hematoxylin–eosin (H&E) staining, followed by light microscopy for morphometric analyses. Similarly, the NRK-52E cells were cultured on coverslips placed in 24-well plates for growth before treatment with 20 μM CP for 4, 8, 14, or 24 h. At the designated time points, the cells were gently washed with PBS, fixed in 4% paraformaldehyde, and subsequently stained with H&E to assess cytopathological changes.

### 2.8. Immunohistochemical Staining Detection After CP Treatment

Deparaffinized and rehydrated sections were incubated overnight at 4 °C with the indicated dilutions of primary antibodies: BAX (1:200), cyt *c* (1:200), NF-κB (1:500), Casp-3 (1:500), Cleaved Casp-3 (1:400), and CryAB (1:100). Following overnight incubation, immunostaining was performed using a standard DAB protocol with an SP rabbit and mouse HRP detection kit (CWBIO, Taizhou, China). Subsequently, the slides were scanned and examined using a digital slide scanner (Grundium Ocus; Tampere, Finland).

### 2.9. TUNEL Apoptosis Assay (TdT-Mediated dUTP Nick-End Labeling) After CP Treatment

To assess apoptotic cell death, transfected NRK-52E cells (either CryAB-silencing or CryAB-overexpressing lines) were seeded onto round coverslips and treated with CP for 12 h. Following three PBS washes, TUNEL assay was performed using a commercially available kit (KeyGEN Bio-TECH). Briefly, cells were fixed and permeabilized, and subsequently fluorescein-labeled via incubation in the reaction mixture (containing Proteinase K, biotin-11-dUTP, and streptavidin-TRITC) provided by the manufacturer. Apoptotic cells were identified by nuclear fluorescence, which indicates DNA fragmentation, and visualized under a fluorescence microscope (Zeiss, Jena, Germany). The ratio of TUNEL-positive nuclei to DAPI-counterstained total nuclei was calculated for statistical analysis.

For kidney tissue sections, the TUNEL assay was performed using the corresponding kit (KeyGEN BioTECH, Nanjing, China). Briefly, deparaffinized and rehydrated sections were rinsed three times in 1× PBS (5 min each) and then incubated with the TUNEL reaction mixture following the kit’s instructions.

### 2.10. Flow Cytometry Analysis After CP Treatment

Cells were seeded into 6-well plates and incubated until they reached 80–90% confluency, and subsequently exposed to 20 μM CP, and harvested after 4, 8, 14, and 24 h. Apoptosis was evaluated with an Annexin V FITC/Propidium Iodide Apoptosis Detection Kit (Vazyme Biotech, Nanjing, China) according to standard procedures [24]. Briefly, cells were washed twice with pre-warmed PBS and detached using 100 μL of trypsin without EDTA for one minute. The trypsin activity was neutralized by adding 1 mL of complete growth medium. The resulting cell suspension was transferred to microcentrifuge tubes and centrifuged at 300× *g* for 5 min at 4 °C. The resulting pellet was washed twice with ice-cold PBS, with each centrifugation repeated under identical conditions. Cells were subsequently resuspended in 100 μL of 1× binding buffer, then stained using 5 μL of Annexin V FITC and 5 μL of propidium iodide, followed by gentle mixing. After a 10 min incubation at room temperature protected from light, 400 μL of 1× binding buffer was added to each sample. Flow cytometry was performed using a BD FACSVerse cell analyzer-Flow cytometer (BD Biosciences, San Jose, CA, USA), and the resulting data were assessed using FlowJo software (version 10.8.1, BD Biosciences, Ashland, OR, USA). A systematic gating hierarchy was established to ensure rigorous cell selection. Initial cell populations were identified, and non-cellular debris was excluded using a primary gate based on forward scatter area versus side scatter area (FSC-A vs. SSC-A). To ensure accurate single-cell quantification, doublets were strictly excluded from analysis using a singlet gate based on forward scatter height versus forward scatter area (FSC-H vs. FSC-A). Apoptotic and necrotic fractions within this verified single-cell population were then resolved using quadrant gating based on Propidium Iodide (PI) and FITC fluorescence intensities. Representative flow cytometry plots reflecting these triplicates from each group are presented.

### 2.11. Western Blotting Analysis After CP Treatment

Kidney tissues were homogenized, and cells were lysed using ice-cold lysis buffer RIPA (Beyotime Biotechnology, Shanghai, China) enhanced with a mixture of protease and phosphatase inhibitors cocktail (MedChemExpress, Monmouth Junction, NJ, USA). The total protein samples, after normalization, were mixed with 5× SDS loading buffer and heated to 95 °C for 5 min. Following SDS-PAGE, proteins were then transferred onto polyvinylidene difluoride (PVDF) membranes (Sigma-Aldrich, St. Louis, MO, USA). The membranes were first blocked using appropriate blocking buffers (either 5% non-fat dry milk, bovine serum albumin, or commercial blocking solution, depending on the manufacturer’s instructions for each specific antibody) and subsequently incubated with primary antibodies against the target proteins along with anti-GAPDH as the internal loading reference control overnight at 4 °C. Immunoblotting was conducted using primary antibodies validated by the manufacturers for specific reactivity with murine tissue/cell targets and handled according to the manufacturers’ protocols. After three washes with Tris-Buffered Saline with Tween 20 (TBST), the membranes were exposed to HRP-linked anti-rabbit or anti-mouse secondary antibodies. Stripping buffer was used to retrieve the membranes between sequential primary antibody incubations for reprobing. Protein signal was developed with an enhanced chemiluminescence (ECL) detection reagent (Thermo Fisher Scientific, Waltham, MA, USA) and captured using a Luminescent Image Analyzer (Amersham ImageQuant 800; Cytiva, Marlborough, MA, USA). Densitometric analysis of the bands was conducted using ImageJ software (Version 1.8.0). Consistent rectangular regions of interest (ROIs) were drawn around target bands, automated background subtraction was applied, and integrated optical densities were mathematically normalized against internal GAPDH loading controls within the same lane before relative expression folds were calculated and plotted. To ensure technical accuracy, each biological sample was analyzed in technical triplicate, with individual targets repeated up to 4 to 5 times to validate densitometric consistency.

### 2.12. Real-Time PCR Analysis Following CP Exposure

RNA was isolated from treated samples with FreeZol reagent (Vazyme, Nanjing, China), according to the manufacturer’s protocol. The obtained RNA was then reverse transcribed into complementary DNA (cDNA) using HiScript Q RT SuperMix Kit (Vazyme, Nanjing, China). Quantitative real-time PCR (qRT-PCR) analyses were performed using SYBR Premix Ex Taq II (TAKARA Bio, Kusatsu, Japan) on an ABI Step One Plus Real-Time PCR System. Gene-specific primers (listed in Table 2) were designed and synthesized by Sangon Biotech (Shanghai, China). The mRNA transcription levels of target genes were quantified by a comparative threshold cycle 2^−ΔΔCt^ method [25], with GAPDH as the housekeeping gene for normalization.

### 2.13. Immunofluorescence Assay After CP Treatment

Immunofluorescence staining was performed to evaluate the expression of apoptosis-related proteins in the renal cells. The cells were seeded on glass coverslips placed in 12-well plates, and subsequently exposed to CP. Following treatment, cells were washed with PBS and fixed with 4% formaldehyde for 20 min at room temperature. After three additional PBS washes, the cells were permeabilized using 0.3% Triton X-100 for 3 min, followed by three subsequent PBS washes. To block non-specific binding sites, the cells were incubated with PBS containing 5% BSA for 1 h at room temperature. The cells were incubated with diluted primary antibody overnight at 4 °C. Following primary antibody retrieval, the samples were washed with PBS and incubated with an FITC-conjugated secondary antibody (Abclonal, Woburn, MA, USA)—either Goat Anti-Rabbit IgG (H + L) or Goat Anti-Mouse IgG (H + L)—at a 1:500 dilution. After three additional PBS washes, the cell nuclei were counterstained using DAPI (Beyotime Biotechnology, Shanghai, China). The coverslips were then mounted onto glass slides using an anti-fade mounting medium (Boster Biological Technology, Wuhan, China) and visualized using a fluorescence microscope (Zeiss, Jena, Germany).

### 2.14. Statistical Analysis

GraphPad Prism version 8.0.2 was used for all statistical analyses. All data are presented as Mean ± SD (*n* = 3 to 6). One-way ANOVA or two-way ANOVA, and Tukey’s multiple comparisons test were used to determine differences between groups. Statistical significance was set at *p* ˂ 0.05. G*power software (version 3.1.9.7) determined that *n* = 6 mice per group was required to maintain sufficient statistical (power 80%, effect size 0.75) to detect significant differences.

## 3. Results

### 3.1. CP Induces Nephrotoxicity in a Dose- and Time-Dependent Manner

**Figure 1:**
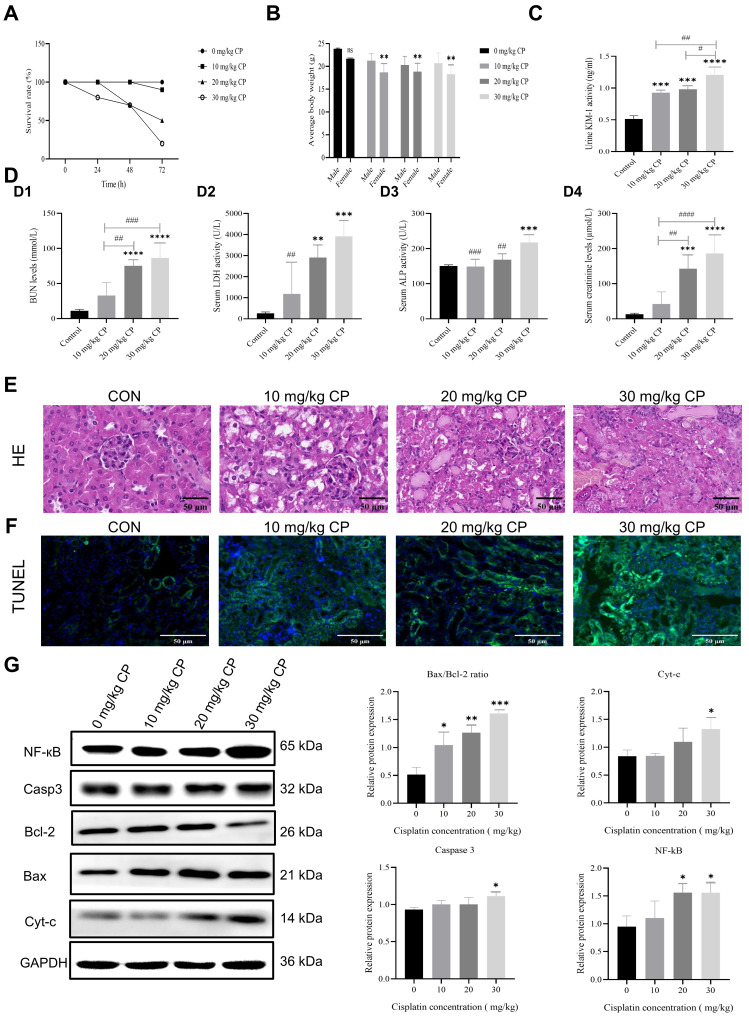
Cisplatin (CP) initiates renal injury in a dose-dependent manner in vivo. Wild-type (WT) C57BL/6J mice received a single i.p. injection of CP (10, 20, and 30 mg/kg) or an equivalent volume of saline. (**A**) Survival curve. (**B**) Body weight changes among experimental groups; *ns*, no statistical significance; ** *p* < 0.01. Changes in renal function marker levels: (**C**) urinary kidney injury molecule-1 (uKIM-1), statistical significance was set at # *p* < 0.05, ## *p* < 0.01, *** *p* < 0.001, and **** *p* < 0.0001, where * vs. control group, and # vs. 30 mg/kg CP group. (**D**) serum levels: blood urea nitrogen (BUN) (**D1**), lactate dehydrogenase (LDH) (**D2**), alkaline phosphatase (ALP) (**D3**), and creatinine (Cr) (**D4**). Statistical significance was set at **/## *p* < 0.01, ***/### *p* < 0.001, and ****/#### *p* < 0.0001, where * vs. control group, and # vs. 30 mg/kg CP group. (**E**) Representative hematoxylin and eosin (HE) staining showing histopathological changes (1 bar = 50 μm, 400×). CP-treated mice displayed progressive lesions, including granular and vacuolar degeneration with loss of epithelial lining at 10 mg/kg CP, prominent protein hyaline deposition and tubular cast formation at 20 mg/kg CP, and a complete loss of renal architecture with extensive tubular epithelial cell necrosis at 30 mg/kg CP. (**F**) Cellular apoptosis detected via TUNEL assay (1 bar = 50 μm, 200×). Representative TUNEL staining sections counterstained with DAPI, showing dose-dependent increases in positive staining within renal tubules, culminating in intense green nuclear fragmentation signaling at 30 mg/kg CP. (**G**) Western blot analysis of apoptosis-related protein levels (cyt *c*, Bax/Bcl-2, Casp-3, and NF-κB) in renal tissues following 72 h of CP treatment. Data are significant at * *p* < 0.05, ** *p* < 0.01, and *** *p* < 0.001.

WT C57BL/6J mice received a single i.p. injection of CP at doses of 0, 10, 20, or 30 mg/kg. Clinical signs in high-dose treatment groups included marked lethargy, reduced motor activity, reduced survival rate (Figure 1A), and fluctuations in body weight (Figure 1B).

To evaluate kidney damage, we performed a biochemical analysis of renal injury markers in mouse serum and urine. The results demonstrated that CP administration induced classic markers of renal injury (Figure 1(D1–D4)), as evidenced by significantly elevated serum levels of BUN, and Cr at a dose of 20 mg/kg CP (BUN, *p* < 0.0001; Cr, *p* = 0.0002); additionally, the tissue damage markers in the mice following CP treatment were assessed by detecting LDH and ALP levels in the serum. Mice treated with CP, LDH enzyme activity (*p* = 0.0059, 20 mg/kg CP), and ALP enzyme activity (*p* = 0.0008, 30 mg/kg CP) in the serum were significantly higher than those of the control group. Meanwhile, CP treatment significantly increased urinary kidney injury molecule-1 (KIM-1) levels in a dose-dependent manner (Figure 1C), peaking at the highest CP dose (*p* < 0.0001), reflecting severe tubular injury.

Histopathological observation of hematoxylin and eosin (H&E)-stained kidney sections revealed that CP caused kidney injury in a dose-dependent manner (Figure 1E). Changes were detectable from 10 mg/kg CP and became pronounced at 30 mg/kg CP. These alterations included glomerular enlargement, dilatation of cortical tubules with loss of epithelial lining, granular and vacuolar degeneration of renal tubular epithelial cells, prominent hyaline protein cast formation within tubular lumina, and marked tubular epithelial cell necrosis characterized by nuclear pyknosis and karyolysis.

Furthermore, CP treatment upregulated the expression of apoptosis-related proteins, including Bax, cyt *c*, Casp-3, and the inflammatory marker NF-κB (Figure 1G). Consistently, the spread of TUNEL-positive signals was significantly increased in renal tissues from CP-treated mice (Figure 1F). Collectively, these data indicate that a dose of 30 mg/kg CP induces the most severe pathological changes, demonstrating that the renal toxicity of CP in mice is dose-dependent.

**Figure 2:**
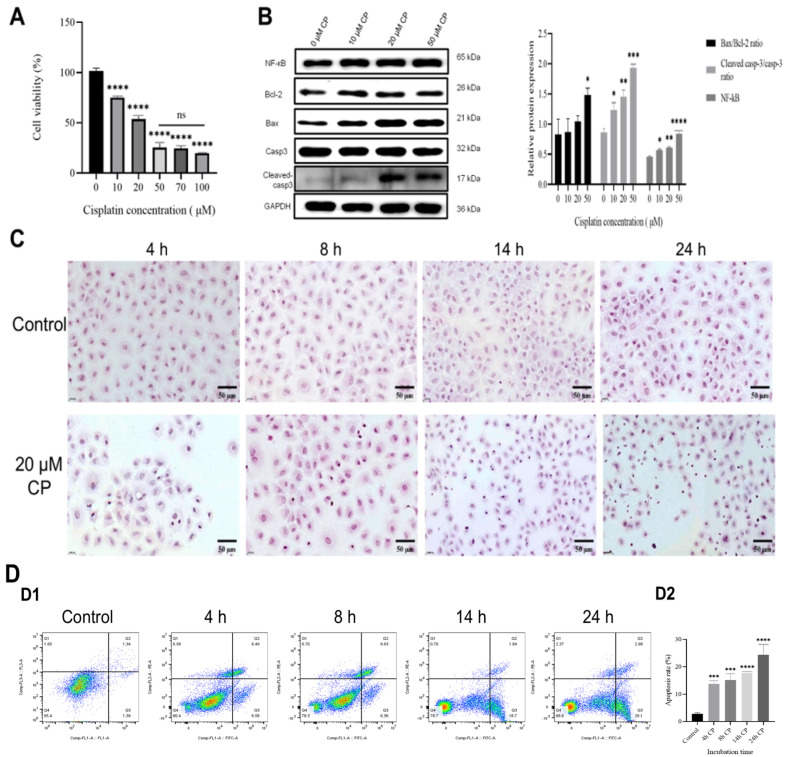
Cisplatin (CP) induces renal injury in a dose- and time-dependent manner in vitro. (**A**) Cell viability of NRK-52E cells measured by the CCK-8 assay following treatment with varying CP concentrations (0, 10, 20, 50, 70, and 100 µM) over 24 h. Data are presented as mean ± SD (**** *p* < 0.0001; ns, no statistical significance). (**B**) Western blot analysis of apoptosis-related protein levels (Bax/Bcl-2, cyt *c*, cleaved Casp-3/Casp-3, and NF-κB) following a 24 h exposure to CP. Statistical significance was set at * *p* < 0.05, ** *p* < 0.01, *** *p* < 0.001, and **** *p* < 0.0001. (**C**) Representative hematoxylin and eosin (HE) staining showing cytopathological changes and cell morphology in NRK-52E cells following treatment with 20 µM CP at various time points (1 bar = 50 μm, 400×). CP-treated cells displayed progressive nuclear hyperstaining at 4 h, vacuolar degeneration and pyknosis at 8 h, cytoplasmic shrinkage and apoptotic features at 14 h, and extensive cell death at 24 h. (**D**) Flow cytometry analysis of cellular apoptosis of NRK-52E cells treated with 20 μM CP over the time course: (**D1**) Representative Annexin V FITC/PI plots, and (**D2**) Quantitative analysis of apoptotic rates. Data are presented as mean ± SD (*** *p* < 0.001 and **** *p* < 0.0001).

To further evaluate the response of renal cells to CP, NRK-52E cells, a rat renal tubular epithelial cell line, were treated with 0, 10, 20, 50, 70, or 100 μM CP for 24 h. Cell viability decreased sharply in a dose-dependent manner, dropping to approximately 50% at 20 μM CP (*p* ≤ 0.0001) (Figure 2A).

H&E staining of NRK-52E cells treated with 20 μM CP for 0, 4, 8, 14, and 24 h revealed no obvious morphological changes in the control group at any time point. After CP exposure, however, early vacuolar degeneration, characterized by cytoplasmic swelling and vacuole formation, was evident at 4 h, indicating initial necrotic changes. By 8 h, many nuclei showed pyknosis, reflecting chromatin condensation typical of late apoptosis or necrosis. At 14 h and 24 h, cells exhibited a marked nuclear condensation and hyperchromasia, loss of cytoplasm, and significant shrinkage, with distinct signs of cell death most pronounced at 24 h (Figure 2C). These morphological alterations indicate that CP triggers cell death in a time-dependent manner.

Consistently, immunoblot analysis revealed that CP upregulated the expression of proteins associated with apoptosis. The Bax/Bcl 2 ratio, NF-κB, and cleavage of Casp-3 expression levels increased significantly after CP exposure (Figure 2B).

Apoptosis was further quantified by flow cytometry (Figure 2D). The basal apoptosis rate in control cells was about 3%; however, CP treatment increased apoptosis to approximately 13%, 15%, 17%, and 29% after 4, 8, 14, and 24 h, respectively (*p* ≤ 0.0006), confirming that CP promotes apoptosis in NRK-52E cells over time.

### 3.2. CP Regulates CryAB Expression in Renal Cells Both In Vivo and In Vitro

**Figure 3:**
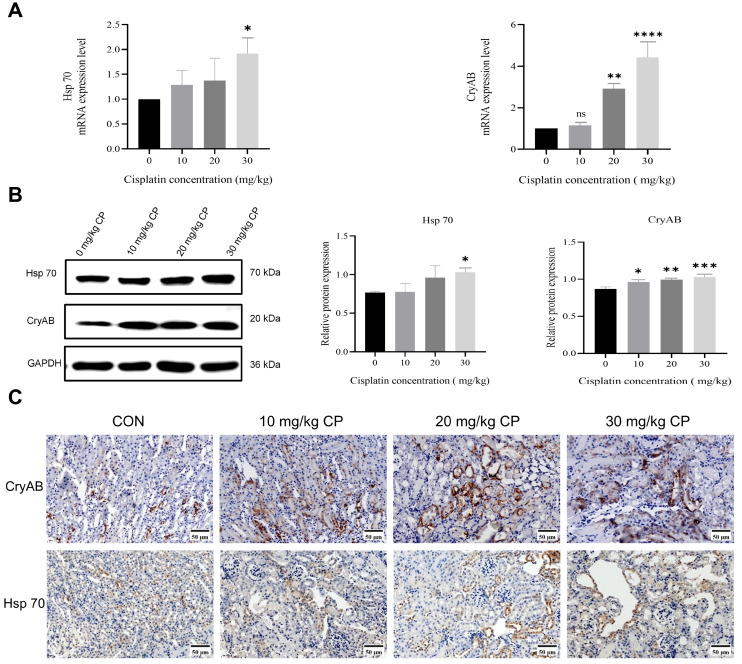
Expression level of renal CryAB in mice after cisplatin (CP) treatment. (**A**) Levels of *Cryab* and *Hsp 70* mRNA measured via RT-qPCR in kidney tissues homogenate from wild-type (WT) mice following CP administration. Statistical significance was set at * *p* < 0.05, ** *p* < 0.01, **** *p* < 0.0001; *ns*, no statistical significance. (**B**) Western blot analysis and relative quantification of CryAB and Hsp 70 protein expression in WT kidney tissues homogenates. Statistical significance was set at * *p* < 0.05, ** *p* < 0.01, and *** *p* < 0.001. (**C**) Immunohistochemistry (IHC) staining of CryAB and Hsp 70 in WT kidney sections following CP treatment (1 bar = 50 μm, 400×).

To assess the role of CryAB in CP-induced nephrotoxicity, we first examined its mRNA expression in kidney tissue homogenates from wild-type mice following CP treatment. The results suggested that CP significantly and dose-dependently altered the transcript levels of *Cryab* (*p* ≤ 0.0001, 30 mg/kg CP), alongside *Hsp 70* (*p* = 0.0372, 30 mg/kg CP) expression (Figure 3A). Consistent with these findings, changes in CryAB and Hsp 70 were also confirmed at the protein level (Figure 3B). Moreover, immunohistochemical (IHC) analysis revealed a marked increase in CryAB protein expression in renal tissues after CP exposure (Figure 3C).

**Figure 4:**
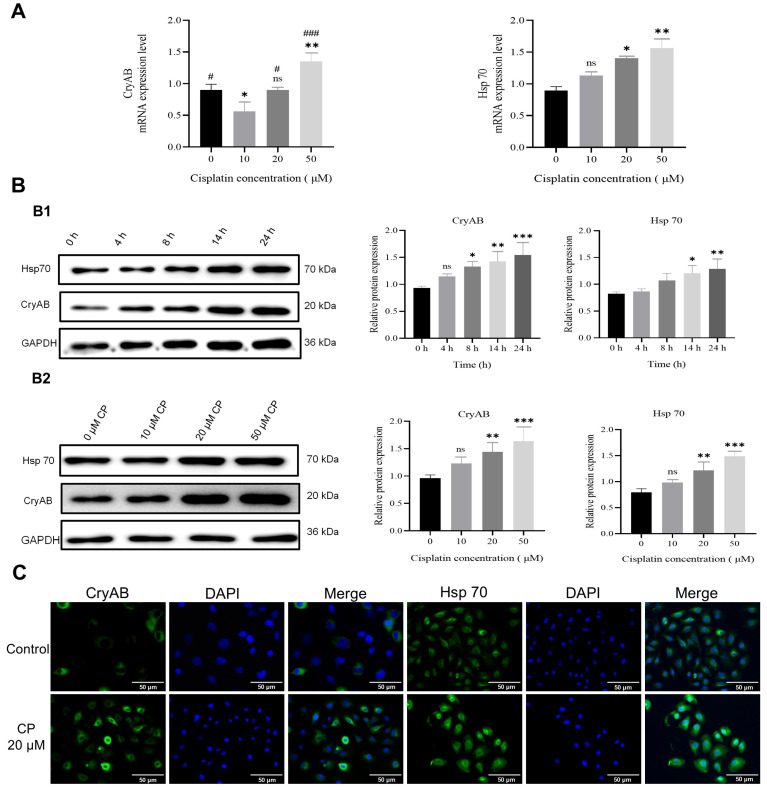
Expression level of CryAB in NRK-52E cells following cisplatin (CP) treatment. (**A**) Levels of *Cryab* and *Hsp 70* mRNA measured via RT-qPCR after 24 h of CP treatment at various concentrations. Statistical significance was set at */# *p* < 0.05, ** *p* < 0.01, ### *p* < 0.001; *ns*, no statistical significance. (**B**) Protein expression of CryAB and Hsp 70 assessed via Western blot following CP exposure: (**B1**) under 20 μM CP at varying time points and (**B2**) after 24 h at different CP doses. Statistical significance was set at * *p* < 0.05, ** *p* < 0.01, *** *p* < 0.001; *ns*, no statistical significance. (**C**) The subcellular localization of CryAB and Hsp 70 visualized via immunofluorescence following treatment with 20 μM CP for 12 h (1 bar = 50 μm, 200×).

Additionally, we evaluated CryAB expression in vitro. Quantitative PCR revealed that CP treatment for 24 h significantly upregulated *Cryab* mRNA in NRK-52E cells in a dose-dependent manner (*p* = 0.0053, 50 µM CP). Similarly, relative mRNA expression of *Hsp 70* demonstrated a significant increase (*p* = 0.0038, 50 µM CP) (Figure 4A). This upregulation was further confirmed at the protein level by immunoblotting, which demonstrated that CryAB expression increased in both a time- (Figure 4(B1)) and a dose-dependent manner (Figure 4(B2)) in CP-treated cells. Similarly, an increase in Hsp 70 expression was observed following CP exposure (Figure 4B). Furthermore, immunofluorescence staining of CryAB and Hsp 70 was visualized by confocal microscopy, demonstrating that CryAB and Hsp 70 proteins were redistributed and accumulated predominantly in the nucleoplasm following CP treatment (Figure 4C).

### 3.3. Silencing of CryAB Accelerates CP-Induced Renal Damage In Vivo and In Vitro

To assess the contribution of CryAB in CP-induced renal damage in vivo, we generated a CryAB KO mouse model using CRISPR/Cas9 technology.

**Figure 5:**
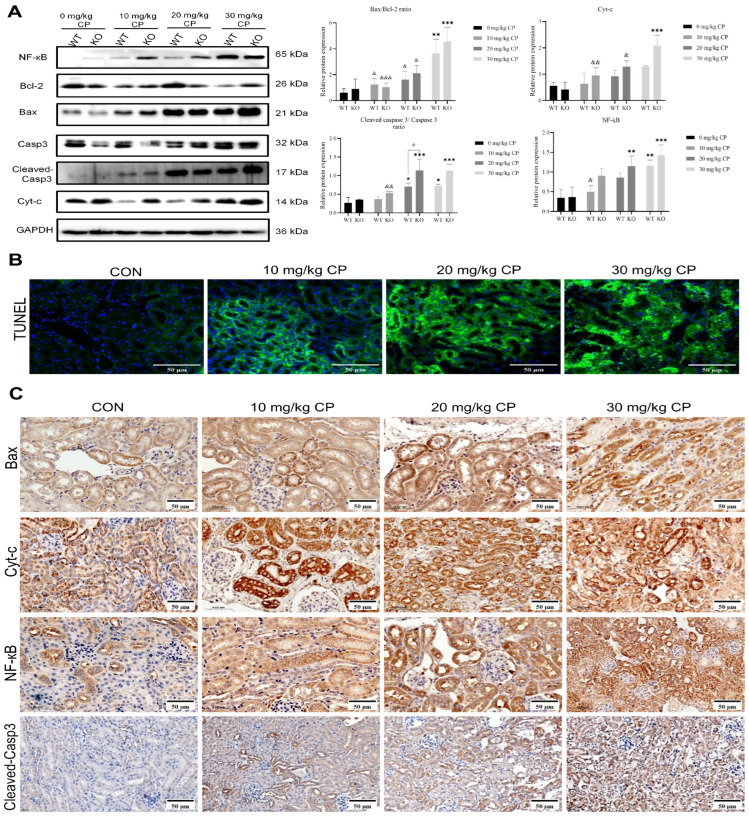
CryAB knockout accelerates cisplatin (CP)-induced renal apoptosis in vivo. (**A**) Apoptosis-related protein levels (Bax/Bcl-2 ratio, cyt *c*, cleaved Casp-3/Casp-3 ratio, and NF-κB) assessed via Western blot in wild-type (WT) and CryAB knockout (KO) mice following a 72 h exposure to specified CP doses. Data were considered significant at */#/& *p* < 0.05, **/&& *p* < 0.01, and ***/&&& *p* < 0.001, where * vs. corresponding control group; # vs. WT and KO groups at an identical CP dose; & vs. 30 mg/kg CP group within their respective genotype (WT or KO). (**B**) Cellular apoptosis evaluated via TUNEL assay (1 bar = 50 μm, 200×). Representative TUNEL staining sections counterstained with DAPI, showing dose-dependent increases in positive staining within renal tubules, culminating in intense nuclear fragmentation signaling at the 30 mg/kg CP dose. (**C**) Immunohistochemical (IHC) analysis of apoptosis-related proteins in renal tissues from KO mice (1 bar = 50 μm, 400×). Representative panels showing staining intensities and localization patterns for Bax, cyt *c*, NF-κB, and cleaved Casp-3 across the indicated CP doses.

Immunoblot analysis of kidney homogenates revealed that, following CP treatment, KO mice exhibited significantly higher levels of key apoptosis-related markers than WT mice. The increase in the ratio of Bax/Bcl 2, cyt *c* expression, the ratio of cleaved Casp-3 relative to total Casp-3, and NF-κB levels occurred in a dose-dependent manner (Figure 5A).

Consistent with these findings, TUNEL staining revealed a marked increase in apoptotic cells in kidney sections from KO mice following CP exposure, which also progressed in a dose-dependent manner (Figure 5B).

IHC analysis further demonstrated altered expression and localization of apoptosis-related markers in KO mice (Figure 5C). Bax expression was elevated and displayed a perinuclear cytoplasmic distribution, correlating with CP dose. Additionally, cyt *c* staining became increasingly diffuse in the cytoplasm following treatment. While cleaved Casp-3 was undetectable in controls, it appeared in numerous nuclei after CP administration and increased with dose. NF-κB, predominantly localized in the cytoplasm, also exhibited enhanced expression with rising CP concentrations.

**Figure 6:**
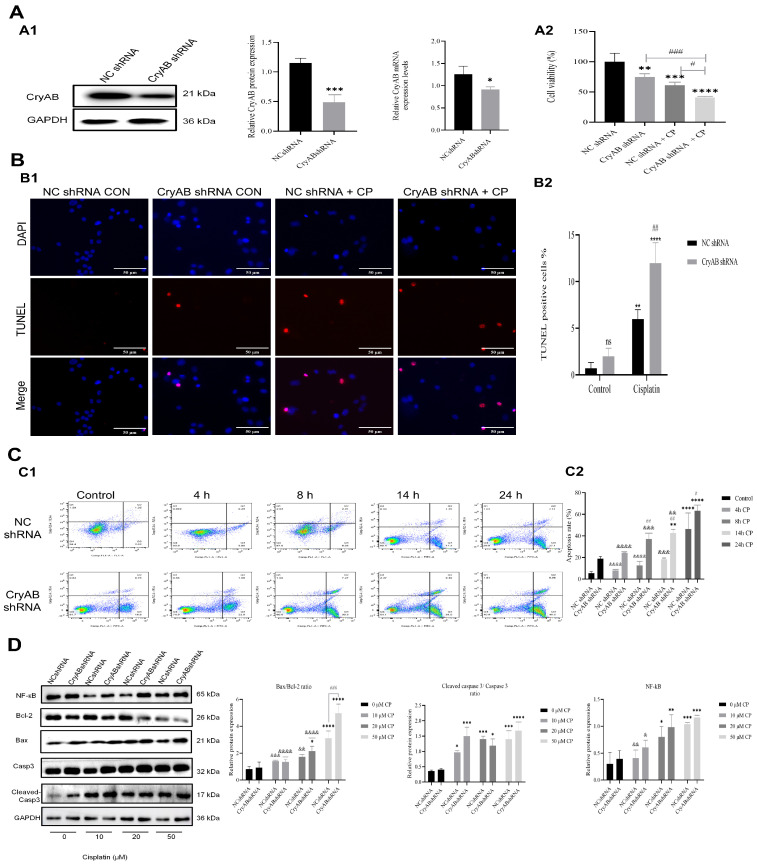
CryAB knockdown accelerates cisplatin (CP)-induced cell apoptosis in vitro. (**A**) Transfection of NRK-52E cells with CryAB shRNA or NC shRNA. (**A1**) Detection of CryAB expression by Western blot and RT-qPCR after gene interference. Statistical significance is denoted as * *p* < 0.05, and *** *p* < 0.001. (**A2**) Cell viability assay of CryAB shRNA (KD) or scramble shRNA (NC) treated with 20 µM cisplatin for 24 h. Significance was set at # *p* < 0.05 and ### *p* < 0.001, ** *p* < 0.01, *** *p* < 0.001 and **** *p* < 0.0001. (**B**) Cellular apoptosis detected via TUNEL assay (1 bar = 50 μm, 200×). (**B1**) Representative panels showing CP-induced nuclear fragmentation labeling (red), which was significantly higher in CryAB shRNA than in NC shRNA controls. (**B2**) Percentage of TUNEL-positive cells. Significance was set at **/## *p* < 0.01, and **** *p* < 0.0001; *ns*, no statistical significance, where * vs. untreated control within the same cell line; # vs. different cell groups under identical treatment. (**C**) Detection of apoptotic rates evaluated by Annexin V FITC/PI flow cytometry assay at indicated time points. (**C1**) Representative plots. (**C2**) Quantitative analysis of the percentage of positive cells. Statistical difference was set at # *p* < 0.05, **/##/&& *p* < 0.01, &&& *p* < 0.001, and ****/&&&& *p* < 0.0001; *ns*, no statistical significance, where * vs. their respective control; # vs. NC shRNA and CryAB shRNA groups at the identical time points; & vs. 24 h CP time point within their respective cell line. (**D**) Expression of apoptosis-related proteins (Bax/Bcl-2, cleaved Casp-3/Casp-3, and NF-κB) measured by Western blot. Statistical significance was set at * *p* < 0.05, ** *p* < 0.01, *** *p* < 0.001, and **** *p* < 0.0001, where * vs. their respective control; # vs. NC shRNA and CryAB shRNA groups under identical treatment; & vs. 50 µM CP group within their respective cell line.

To further investigate the function of CryAB in vitro, NRK-52E cells were stably transfected either with CryAB-targeting shRNA (knockdown, KD) or with non-targeting scramble shRNA (negative control, NC) to generate cell lines for comparison. Western blot analysis confirmed a significant reduction in CryAB protein in KD cells compared with NC cells (Figure 6(A1)). Following CP treatment (20 µM), the viability of CryAB knockdown cells was markedly decreased compared to the scrambled control cells at the corresponding dose and time point (*p* = 0.0182) (Figure 6(A2)).

TUNEL staining revealed that CryAB knockdown was associated with enhanced CP-induced apoptosis in NRK-52E cells (Figure 6B). Following CP exposure, TUNEL-positive signals were observed in both NC and KD cells, with a notably pronounced rate in the KD group compared to the NC group at the corresponding dose and time point (*p* = 0.0031). This result was further supported by flow cytometry: following the knockdown of CryAB, the apoptotic rates at 0, 4, 8, 14, and 24 h post CP treatment reached approximately 16%, 24%, 37%, 43%, and 57%, respectively, each significantly higher than that in the NC group at the corresponding time point (Figure 6C).

At the protein level, silencing of CryAB was accompanied by an increase in the ratio of Bax/Bcl-2 and the ratio of cleaved Casp-3/Casp-3 in renal cells treated with CP (Figure 6D). Moreover, CryAB inhibition resulted in elevated expression of NF-κB in CP-exposed cells compared with controls. Together, these data suggest that the loss of CryAB function is associated with an increased CP-induced renal cellular injury.

**Figure 7:**
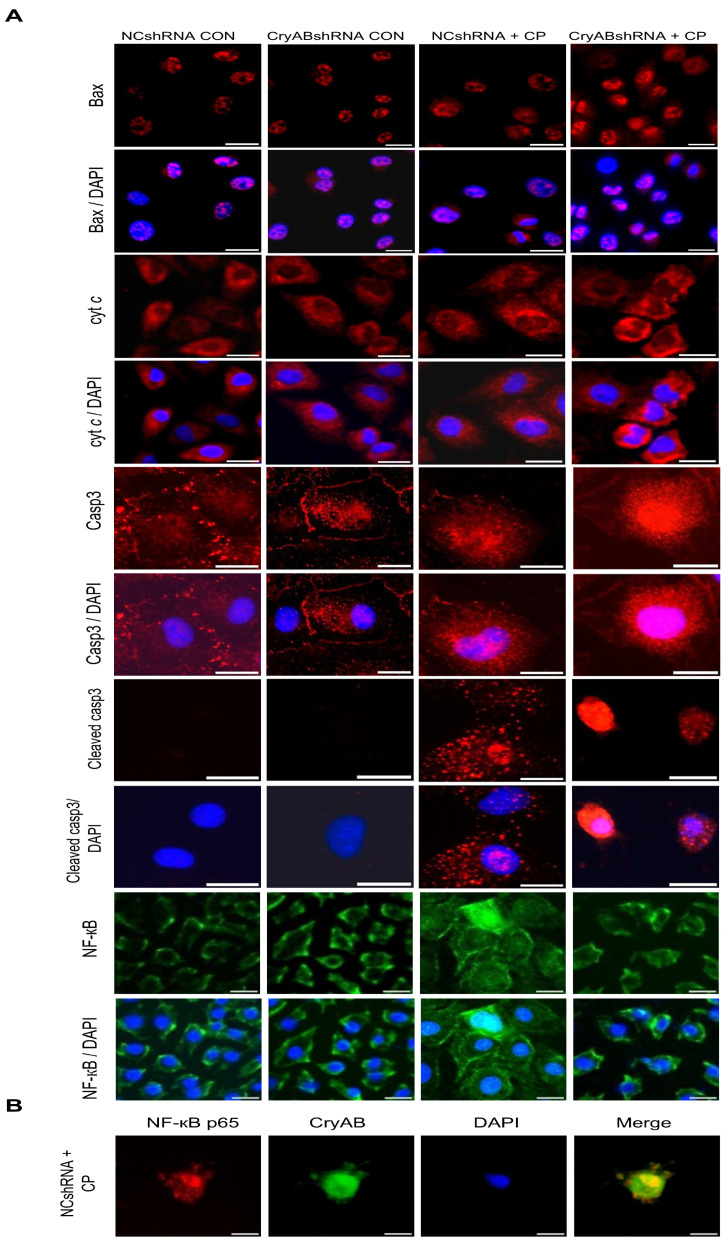
Localization of proteins associated with apoptosis in NCshRNA cells and CryABshRNA cells following cisplatin (CP) treatment. Cells were treated with CP (20 µM) for 12 h and then fixed, permeabilized, and immunofluorescence was performed to check protein expression and localization of (Bax, cyt *c*, Casp-3, cleaved Casp-3, and NF-kB) using fluorescent microscopy. (**A**) Representative channels of target protein signals (green/red), nuclei counterstained with DAPI (blue), and merged images are shown (1 bar = 10 μm, 400×). Following CP treatment, Bax immunofluorescence accumulated at the nuclear periphery of apoptotic cells; many CryABshRNA cells exhibited a loss of mitochondrial cyt *c*, resulting in diffuse cytosolic staining; active caspase-3 fluorescence increased and translocated to the nucleus, enabling detection of the cleaved Casp-3 subunit; lack of nuclear translocation for the NF-κB subunit p65 was observed in CryABshRNA cells. (**B**) Co-immunostaining of NF-κB and CryAB proteins in NCshRNA cells following CP treatment (1 bar = 10 μm). Representative channels of target protein signals (green/red), nuclei counterstained with DAPI (blue), and merged images are displayed.

The expression and localization of apoptosis-related proteins, including Bax, cyt *c*, Casp-3, cleaved Casp-3, and NF-κB, were examined by indirect immunofluorescence in NC shRNA and CryAB shRNA cells following CP treatment (Figure 7A).

In control cells of both groups, Bax displayed uniform, diffuse nuclear localization with a weaker cytoplasmic signal. Following CP exposure, particularly in CryAB shRNA cells, Bax immunofluorescence partially redistributed to the nuclear periphery in apoptotic cells.

Additionally, cyt *c* exhibited a punctate, perinuclear mitochondrial pattern in untreated cells. Following CP treatment, many CryAB shRNA cells lost mitochondrial cyt *c* staining, instead displaying faint, diffuse cytosolic fluorescence.

Furthermore, Casp-3 was diffusely localized in the cytoplasm of control cells, with no cleaved form detected. Following CP treatment, Casp-3 signal increased and accumulated in the nucleus, and cleaved Casp-3 became apparent, predominantly in CryAB shRNA cells.

NF-κB in untreated cells was mainly cytoplasmic, appearing as a diffuse or granular stain. CP induced limited nuclear translocation of NF-κB in CryAB shRNA cells compared to NC shRNA cells, suggesting that CryAB interference restricts NF-κB nuclear entry. This suggests that CP may modulate NF-κB transcriptional activity for cell survival, and CryAB may influence this effect by facilitating NF-κB nuclear translocation.

To further validate NF-κB activity, co-immunostaining of NF-κB p65 and CryAB was performed in CP-treated NC shRNA cells. The results revealed co-localization of CryAB with NF-κB p65 in cells undergoing CP-induced nuclear translocation of NF-κB (Figure 7B).

### 3.4. CryAB Overexpression Attenuates CP-Induced Injury in NRK-52E Cells

**Figure 8:**
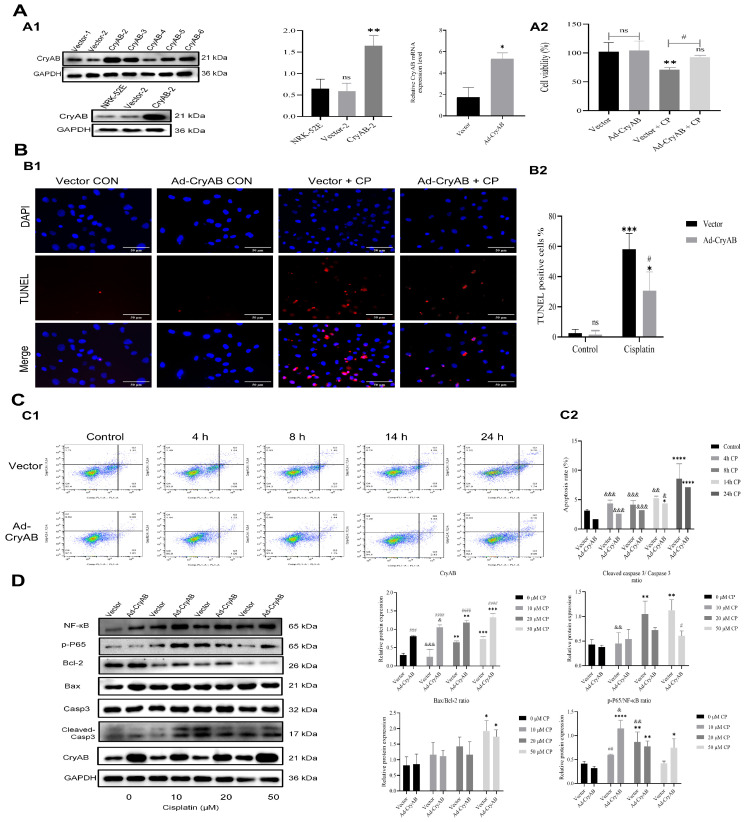
CryAB overexpression mitigates cisplatin (CP)-induced injury in vitro. (**A**) Stable transfection of CryAB overexpression plasmid in NRK-52E cells. (**A1**) Detection of CryAB protein and mRNA expression levels in Vector and Ad-CryAB cells after transfection using Western blot and quantitative real-time PCR (qRT-PCR) analyses, respectively. Data are significant at * *p* < 0.05, ** *p* < 0.01; *ns*, no statistical significance. (**A2**) Cell viability assay of Vector and Ad-CryAB cells treated with 20 µM cisplatin for 24 h. Statistical difference set at # *p* < 0.05 and ** *p* < 0.01, where * vs. respective control, and *ns* denotes non-statistical significance. (**B**) Cellular apoptosis in Vector and Ad-CryAB cells following CP treatment detected via TUNEL assay (1 bar = 50 μm, 200×). (**B1**) Representative panels showing CP-induced nuclear fragmentation labeling, which was significantly lower in Ad-CryAB cells than in Vector cells. (**B2**) Percentage of TUNEL-positive cells. Data were considered significant at */# *p* < 0.05, *** *p* < 0.001; *ns*, no statistical significance, where * vs. control within the same cell line; # vs. cell groups under identical treatment. (**C**) Detection of apoptotic rates by Annexin V-FITC/PI flow cytometry assay at indicated time points. (**C1**) Representative plots. (**C2**) Quantitative analysis of the percentage of positive cells. Statistical difference was set at */& *p* < 0.05, && *p* < 0.01, &&& *p* < 0.001, and **** *p* < 0.0001, where * vs. their respective control; & vs. 24 h CP time point within their respective cell line. (**D**) Expression of apoptosis-related proteins (Bax/Bcl-2, cleaved Casp-3/Casp-3, and NF-κB) measured by Western blot. Statistical significance was set at */#/& *p* < 0.05, **/##/&& *p* < 0.01, ***/###/&&& *p* < 0.001, and ****/#### *p* < 0.0001, where * vs. their respective control; # vs. Vector and Ad-CryAB groups under identical treatment; & vs. 50 µM CP group within their respective cell line. Note: The prefix “Ad-” in the figure panels serves as an abbreviation for “Added recombinant pc-DNA3.1+ for CryAB overexpression”.

To determine whether CryAB overexpression can attenuate CP-induced cytotoxicity in renal cells, we stably transfected NRK-52E cells with a recombinant pcDNA3.1+ expression vector containing the target *Cryab* gene sequence (Ad-CryAB), while an empty vector served as a vector control (VC). Stable clones were selected using zeocin, and both the mRNA and protein expression levels of CryAB were confirmed to be significantly elevated in Ad-CryAB-transfected cells (*p* = 0.0048) compared with VC cells (Figure 8(A1)).

CryAB overexpression markedly improved cell viability following CP treatment (*p* = 0.4092), mitigating the reduction observed in vector cells (*p* = 0.0032) (Figure 8(A2)). TUNEL assay further revealed that, although CP increased apoptosis in both groups, the number of TUNEL-positive cells was significantly lower in CryAB-overexpressing cells (*p* = 0.0115) than in vector cells (*p* = 0.0002) (Figure 8B). Consistently, flow cytometry analysis showed that CP-induced apoptosis increased over time in VC cells, whereas CryAB overexpression substantially reduced this apoptotic response (*p* ≤ 0.0001). At 24 h post-CP treatment, the apoptotic rate was decreased to approximately 7% compared to 12% in VC cells (Figure 8C).

Western blot analysis suggested that CP increased the expression of pro-apoptotic markers, including Bax/Bcl-2 ratio and the ratio of cleaved Casp-3 to total Casp-3 in VC cells. These changes were significantly attenuated by CryAB overexpression (Figure 8D). Moreover, the expression of pP65, a key subunit of activated NF-κB, was elevated in Ad-CryAB cells following CP exposure. This suggests that CryAB may promote cell survival under CP stress, at least in part, through modulation of the NF-κB pathway.

### 3.5. CryAB Knockdown Exacerbates CP Activation of the MAPK Pathway in Renal Cells

**Figure 9:**
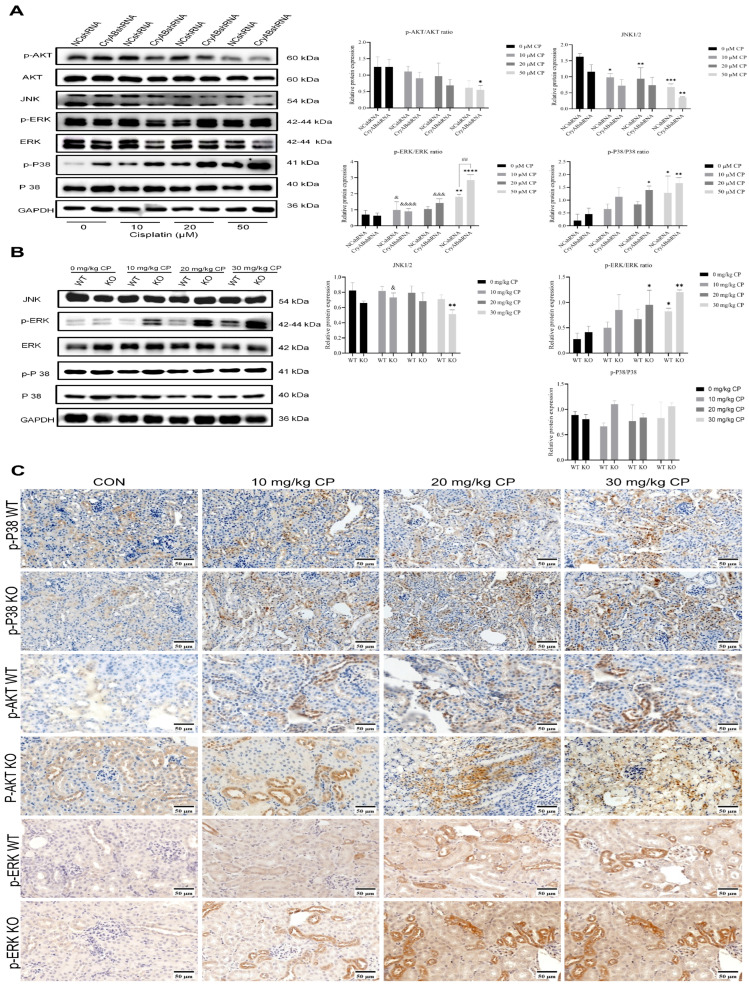
Expression of MAPK signaling components in CryAB knockdown renal cells following cisplatin (CP) treatment. (**A**) Immunoblot analysis of MAPK signaling components in αBshRNA and NCshRNA cells following a 24 h treatment with specified CP concentrations. Statistical significance is denoted as */& *p* < 0.05, **/## *p* < 0.01, ***/&&& *p* < 0.001, and ****/&&&& *p* < 0.0001, where * vs. corresponding control group; # vs. NCshRNA and CryABshRNA groups at identical CP doses; & vs. 50 µM CP treatment group within their respective cell line. (**B**) Western blot analysis of MAPK signaling components in kidney tissues from wild-type (WT) and CryAB knockout (KO) mice following CP administration. Statistical significance was set at */& *p* < 0.05, and ** *p* < 0.01, where * vs. respective control group; & vs. CP 30 mg/kg group of their respective genotype. (**C**) Immunohistochemical (IHC) analysis of MAPK signaling components in renal tissues from WT and KO mice following CP treatment (1 bar = 50 μm, 200×). Representative kidney tissue sections showing p-P38, p-AKT, and p-ERK staining; dose-dependent increases in staining intensity and nuclear translocation were observed for all three proteins following CP treatment, with these changes being markedly more pronounced in KO mice than in WT controls.

This study examined the modulatory role of CryAB in MAP kinase activation during CP-induced nephrotoxicity. Immunoblot analysis revealed that in CP-treated NRK-52E cells, knockdown of CryAB was associated with increased phosphorylation of ERK and p38 in a dose-dependent manner, compared with NC shRNA cells, whereas JNK1/2 activation under the same conditions was modest (Figure 9A). Additionally, baseline AKT phosphorylation was notably higher in NC shRNA cells compared to CryAB-deficient cells, and this phosphorylation progressively declined with escalating doses of CP.

Consistent with the in vitro findings, kidney tissues from CP-treated KO mice exhibited markedly higher phosphorylation of MAPK signaling cascades (p-ERK1/2, p-P38) compared to WT mice receiving an identical CP dose (Figure 9B).

IHC staining further corroborated these findings. In KO mice, CP treatment induced a dose-dependent increase in nuclear translocation of p-ERK1/2 and p-P38 in renal tissues, an effect that was markedly more pronounced than that observed in WT mice, whereas the AKT pathway exhibited a progressive decline in phosphorylation with escalating CP doses (Figure 9C). Together, these data suggest that the absence of CryAB is associated with enhanced CP-induced MAPK phosphorylation while reducing AKT phosphorylation both in vitro and in vivo.

## 4. Discussion

CP, a platinum-based inorganic complex, is widely used to treat various malignant tumors. A major adverse effect of CP chemotherapy is nephrotoxicity, which often manifests as toxic acute kidney injury (AKI) [18]. In both humans and experimental animals, CP accumulates preferentially in the kidneys and induces damage primarily in epithelial cells, especially within the S3 portion of the proximal tubule [26]. CP-induced nephrotoxicity results from the interplay of multiple signaling pathways linked to apoptosis and other forms of cell death [7].

CryAB, a small heat shock protein with multifaceted functions, exhibits complex and context-dependent biological effects, frequently mediated through phosphorylation-dependent mechanisms [14]. Although Hsps serve a cytoprotective role in stressed animals [27], elevated expression of CryAB in numerous cancers is frequently linked with tumor aggression and poor prognosis, highlighting a critical paradox [15]. Under cellular stress or injury, renal cells trigger protective responses, including the heat shock pathway, to defend against stress, in which the defensive and adaptive pathway is studied in models of ischemic kidney injury [28]. CryAB is expressed in healthy renal epithelia and, given its chaperone and anti-apoptotic activities, is regarded as a cell protective agent in the hyperosmotic environment of the renal medulla [10]. The activation of the heat shock protein network is critical during renal stress, as evidence suggests that HSF1 deficiency impairs CryAB expression and subsequently exacerbates susceptibility to CP-induced nephrotoxicity [29]. In renal cell carcinomas, upregulation of CryAB disrupts cell adhesion, promoting distant metastasis and invasive potential [30,31]. CryAB significantly influences cellular stress responses and cell survival. For example, CryAB plays a crucial role in promoting TGF β signaling, which represents a critical mechanism in pathological tissue remodeling and disease progression [32]. Conversely, in colorectal cancer, CryAB overexpression correlates with poor prognosis and prompts epithelial–mesenchymal transition (EMT) and tumor cell invasion through the activation of ERK signaling [33]. Silencing CryAB has been reported to inhibit metastatic potential in mouse models and to sensitize breast cancer cells to apoptosis [15,32].

The involvement of the cellular heat shock response in CP-induced renal toxicity has been examined in prior studies, yet it remains unclear to what extent renal apoptosis—a well-established consequence of CP nephrotoxicity [34]—is modulated by CryAB, and the exact underlying mechanisms are yet to be fully elucidated. Although CryAB is conventionally implicated in the broader cellular response to stress, its specific regulation during CP-induced injury appears indirect and multistaged. Therefore, our study aimed to clarify the functional role of CryAB and its corresponding molecular mechanisms in this context.

Using renal epithelial cells from mice and rats as experimental models, this work demonstrates that the small heat shock protein CryAB is activated in the context of CP-induced kidney damage, a response accompanied by induction of apoptotic markers and MAP kinases. Nonetheless, CryAB exerted a cytoprotective effect in renal tubular cells, partly through effectors within the apoptotic pathway. Additionally, CP administration in mice caused marked kidney dysfunction, evidenced by histopathological evaluation of kidneys in our animal model, which revealed that a low dose of CP led to apoptosis, while a high dose of CP caused necrosis.

In the present study, the renal tissue damage induced by CP was evidenced by significant elevations in urinary KIM-1 levels, serum Cr, and BUN, alongside increased serum activities of LDH and ALP. These changes indicate that CP-induced cellular and tubular damage compromises the filtration barrier, releasing injury-associated enzymes into the bloodstream, which is consistent with prior research stating that kidney injury molecule 1, an early diagnostic biomarker of CP nephrotoxicity [35], exhibited anti-inflammatory properties after CP or ischemia-induced acute kidney injury via interaction with p85 and PI3K-dependent downregulation of NF-κB [36].

Renal tubules are the primary site of CP-induced injury and apoptosis. Tubular apoptosis is driven largely by the intrinsic pathway, involving mitochondrial Bax accumulation, cyt *c* release, and Casp-3 cascade activation. Heat shock proteins, including CryAB and HSP20, participate in mitochondrial apoptosis; for instance, CryAB inhibits Casp-3 and -8 maturation and activation after TNF-α treatment [37]. Using CryAB knockout and wild-type mouse models, we found that CryAB deletion sensitized renal cells to CP-induced apoptosis and exacerbated kidney injury in vivo, which matched with in vitro knockdown findings, which revealed that CryAB knockdown in NRK-52E cells elevated proteins that promoted apoptosis (Bax, cyt *c*, Casp-3) and cleavage of Casp-3. Conversely, CryAB overexpression attenuated renal damage, enhanced P65 phosphorylation, and reduced Casp-3 cleavage, thereby partially suppressing mitochondrial apoptosis. Generally, CP nephrotoxicity was believed to activate several survival-related pathways, such as NF-κB, which mediates both pro- and anti-apoptotic responses [38]. Our data revealed a distinct distribution pattern between CryAB and NF-κB pathways during CP stress; our co-immunostaining assay demonstrated a nuclear colocalization of CryAB and NF-κB following CP treatment in renal cells, whereas NF-κB remained primarily sequestered in the cytoplasm of CryAB-deficient cells. While these findings suggest that CryAB presence influences nuclear translocation or cytoplasmic retention of NF-κB, concluding a definitive functional switch between pro-survival and pro-apoptotic NF-κB would be speculative and lacks upstream mechanistic validation. Consequently, we limit our current interpretation to the subcellular localization pattern and protein expression changes in p-P65, guiding future functional validation of this axis.

Similarly, MAPK phosphorylation regulates proliferation, stress response, and survival [39] and contributes to CP nephrotoxicity [40,41]. Our data suggested a potential regulatory interplay between MAPK signaling and CryAB expression during CP exposure. We observed that CP-induced phosphorylation of ERK1/2 and p38 MAPK mirrors changes in CryAB expression. The marked elevation of p38 phosphorylation observed in CryAB-deficient models suggests that this signaling axis may contribute to CP-induced injury. This finding aligns with previous reports demonstrating that p38 can serve as a potent pro-apoptotic mediator; for instance, it has been shown to phosphorylate p68 at T564/T446 to promote apoptosis in oxaliplatin-treated colon cancer cells [42]. The AKT signaling pathway plays an essential role in the progression of cell death and inflammation. For instance, its upregulation has been shown to promote hepatic carcinoma development. Once activated, AKT inhibits pro-apoptotic proteins like Bad and regulates NF-κB signaling via phosphorylation of IKKα [43,44]. PI3K/AKT inhibition has also been demonstrated to sensitize ovarian cancerous cells to CP and enhance its anti-tumor efficacy in xenograft models [45]. Our established genetic models demonstrated that altering CryAB expression levels directly shifts the phosphorylation profiles of MAPK and AKT signaling pathways during CP stress. Through parallel in vivo gene knockout and in vitro expression models, we observed a synchronized increase in stress-responsive MAPK phosphorylation, whereas pro-survival AKT signaling exhibited a progressive depletion. This pattern was further supported by a dose-dependent nuclear translocation of p-ERK and p-P38 in renal tissues of KO mice. However, our current study design did not utilize specific pharmacological inhibitors or targeted genetic silencing of each downstream cascade. Therefore, we cannot determine the exact degree to which each of these components drives the overall protective phenotype. We focused our interpretation on synchronized modulation of these pathways with CryAB, rather than a causative mechanism of a single downstream signaling cascade.

Clarifying all the mechanisms involved may lead to strategies to reduce CP-induced side effects. While prior studies established the fundamental cytoprotective role of the HSF1/CryAB axis against direct tubular apoptosis [11], here our findings expand this scope and provide strong genetic evidence supporting a functional role for CryAB in this process. By utilizing an in vivo CryAB knockout model alongside in vitro gene overexpression and knockdown in renal cells, we demonstrate that CryAB acts as a vital upstream coordinator of renal cell survival. Specifically, its presence or absence dictates the synchronized regulation of mitochondrial apoptosis, NF-κB, MAPK, and AKT pathways; these findings reveal that CryAB regulation does not operate independently, but rather commands an integrated molecular network response that clarifies the mechanistic contribution of CryAB in mitigating CP nephrotoxicity. Nevertheless, a limitation of this study is that direct pathway-specific causality was not established using pharmacological inhibitors. Future studies will focus on utilizing specific pathway blockers to delineate these causal mechanisms fully.

## 5. Conclusions

In conclusion, this study provides a genetically validated framework for understanding the potential protective mechanisms of CryAB in CP-induced renal injury. Through parallel in vivo and in vitro genetic manipulation strategies, our findings suggest that CryAB may act as an important regulatory component, modulating mitochondrial apoptosis and associated survival pathways. The alignment of our data indicates that CryAB provides a multifaceted protection under these experimental conditions, offering valuable molecular insights that may be useful for mitigating CP-induced nephrotoxicity.

## Figures and Tables

**Table 1:** Sequences for the shRNA oligonucleotide sequences.

Gene	Sense	Antisense
shRNA-1	5′-GGUGCACGGCAAGCACGAATT-3′	5′-UUCGUGCUUGCCGUGCACCTT-3′
shRNA-2	5′-CAGAGGAACUCAAAGUCAATT-3′	5′-UUGACUUUGAGUUCCUCUGTT-3′
shRNA-3	5′-GAGUCUGACCUCUUCUCUATT-3′	5′-UAGAGAAGAGGUCAGACUCTT-3′

**Table 2:** The primer sequences utilized for quantitative real-time PCR.

Gene	Forward Primer	Reverse Primer	Accession Number
	Mouse		
*Gapdh*	CCTCGTCCCGTAGACAAAAT	ACAATCTCCACTTTGCCACT	NM_008084
*Cryab*	TGTTTCTCTTTTCTTAGCTCAAAGC	TGAGAAGAGGTCAGACTCCA	NM_009964
*Hsp70*	CCTGCAGAATTCGACCTACA	AGCAGGGTTAGAAATCCAGAC	NM_010479
	Rat		
*Gapdh*	CTCTGCTCCTCCCTGTTCTA	ATACGGCCAAATCCGTTCA	NM_017008
*Cryab*	GACCTCTTCTCTACAGCCAC	GATGAAGCCATGTTCGTCCT	NM_012935
*Hsp70*	GCTCCTTTCTCCAAAGTCCT	GTCCACCTGCATCTTCTCTT	NM_031971

## Data Availability

The datasets generated or analyzed during the current study are available from the corresponding author upon reasonable request.
